# Supplementary material for: The genetic basis for the adaptation of E. coli to sugar synthesis from CO2
Source: Nat Commun. 2017 Nov 22;8:1705. doi: 10.1038/s41467-017-01835-3 (PMC5700066; doi:10.1038/s41467-017-01835-3)
Supplement: Supplementary file 1 — Supplementary Information [file 41467_2017_1835_MOESM1_ESM.pdf]

<u>Supplementary Information</u>

(a)

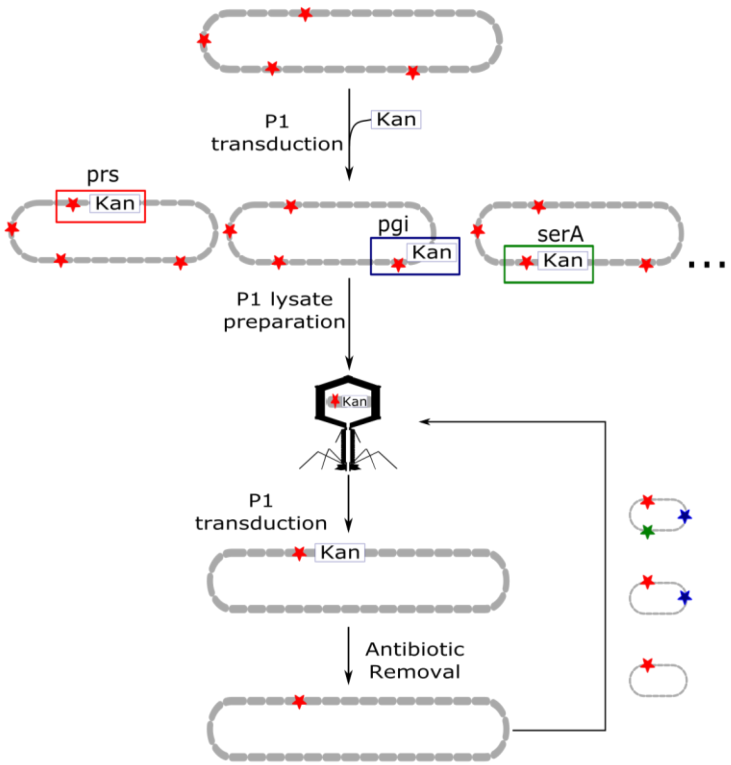

(b)

Mutations / Number	prs	ΔppsR	serA	pgi	crp	malT	xylA	glmU	pyrH	Hemi. Growth
rep.1	★	★	★	★	★	★	★	★		✓
rep.2	★	★	★	★	★	★	★		★	✓
#1		★								X
#2	★									X
#3	★		★							X
#4	★			★						X
#5	★		★	★						X
#6	★		★	★	★					X
#7	★		★	★		★				X
#8	★	★								X
#9	★	★	★							X
#10	★	★		★						X
#11	★	★	★	★						X
#12	★	★	★	★		★				X
#13	★	★	★	★	★					✓

Supplementary Figure 1. Genetic Reconstruction using multiple P1 transduction. (a) To transfer a mutation from the evolved strain to a new genetic background, we chose an adjacent gene that will serve as a selection marker ("helper gene"). We prepared a lysate of a KEIO collection strain with a kanamycin resistance cassette inserted instead of the helper gene. The evolved strain was then transfected with this lysate and plated on kanamycin plates. We selected for colonies which contained the resistance cassette and also the mutation of interest. We prepared lysates from those colonies and transfected the target cells with it. The close proximity of the selection marker to the mutation of interest insured that target cells which are resistant to kanamycin will have high probability to contain the mutation of interest. We screened for successful transfer of the mutation of interest to the target cells. In all iterations excluding the last transduction, the antibiotics resistance marker was removed by the pCP20–flp system in order to enable repeated usage for selection of next desired allele. (**b)** Different combinations of mutations originated from evolution "replay" strains (noted here as rep. 1 and rep. 2) were explored to find the smallest set. For each set, hemiautotrophic growth was tested in liquid after transforming with pCBB plasmid containing *RuBisCO* and *prk*. We chose to test mutations starting from branch points enzymes and regulators, hypothesizing that the mutations that appeared in one but not both 'replay isolated strains' are not required and that the *xylA* mutation is not essential for the final phenotype. As can be observed we also found that the *malT* mutation was not needed. In total 13 strains were constructed and tested for hemiautotrophic growth until the phenotype was reproduced.

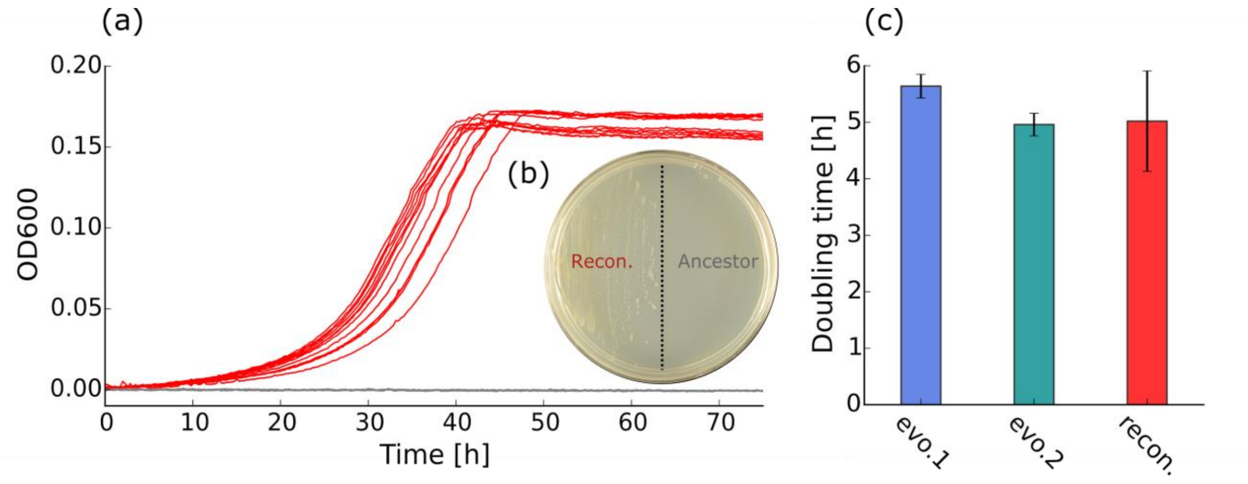

Supplementary Figure 2. Characterization of the hemiautotrophic phenotype of the reconstructed hemiautotrophic strain. The reconstructed hemiautotrophic strain was able to grow on pyruvate as a sole organic carbon source both in liquid (a) and on agar plates (b), in contrast to the ancestral strain. In both cases, growth required elevated CO_2 conditions ($pCO_2 = 0.1$ atm) and no growth was detected under ambient atmosphere. (c) The reconstructed hemiautotrophic strain has a doubling time *(mean ± SD; n=3)* similar to that of the chemostat evolved strains (≈5hr doubling time).

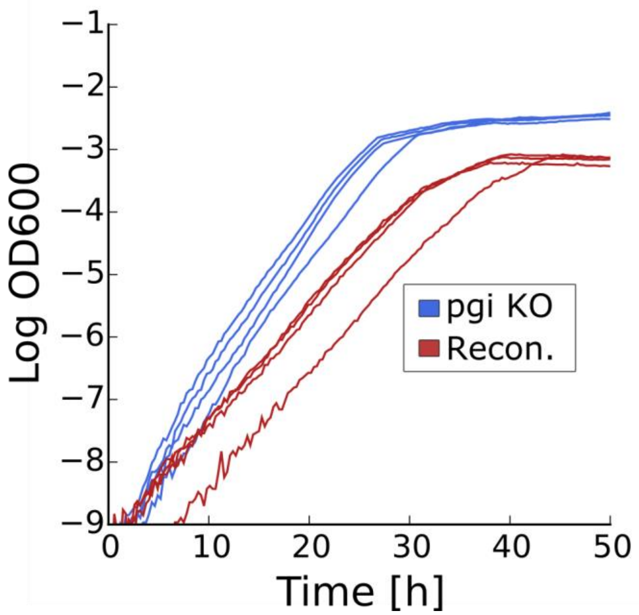

Supplementary Figure 3. The *pgi* deletion (Δ*pgi*) is sufficient to complement the original *pgi* mutation present in the reconstructed strain (*pgi*:G378C). Replacing the original *pgi* mutation in the reconstructed hemiautotrophic strain with a knockout allele enabled the cells to grow hemiautotrophically. We interpret this to indicate that a decrease in the amount of flux from F6P to G6P is required for hemiautotrophic growth.

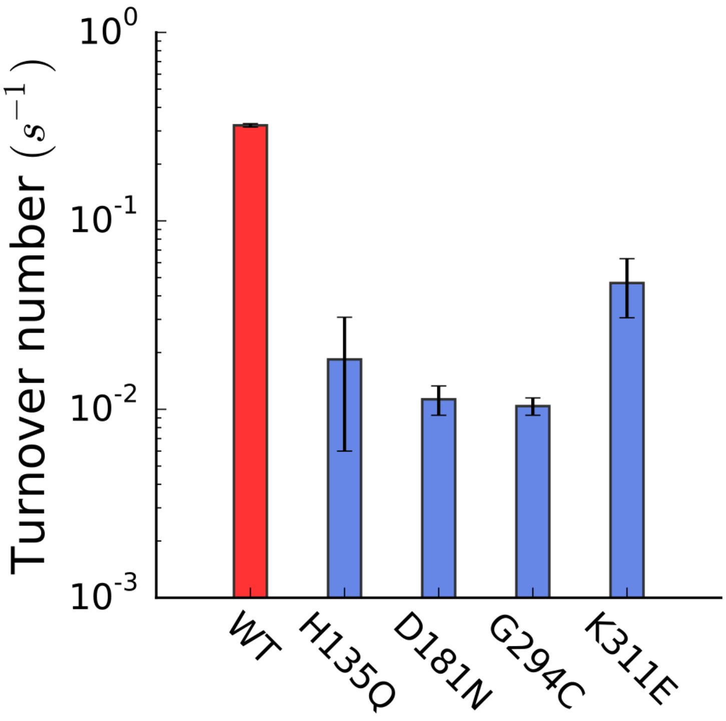

Supplementary Figure 4. *in vitro* activity assay for WT and *SerA* mutants. Measurements were performed by spectroscopically following the decrease of NADH during the reduction of 2-oxoglutarate. The levels of NADH were quantified by measuring absorbance at 340nm *(mean ± SD; n=3)*. The assay was done at saturating levels of NADH 0.8mM and 1mM 2-oxoglutarate.

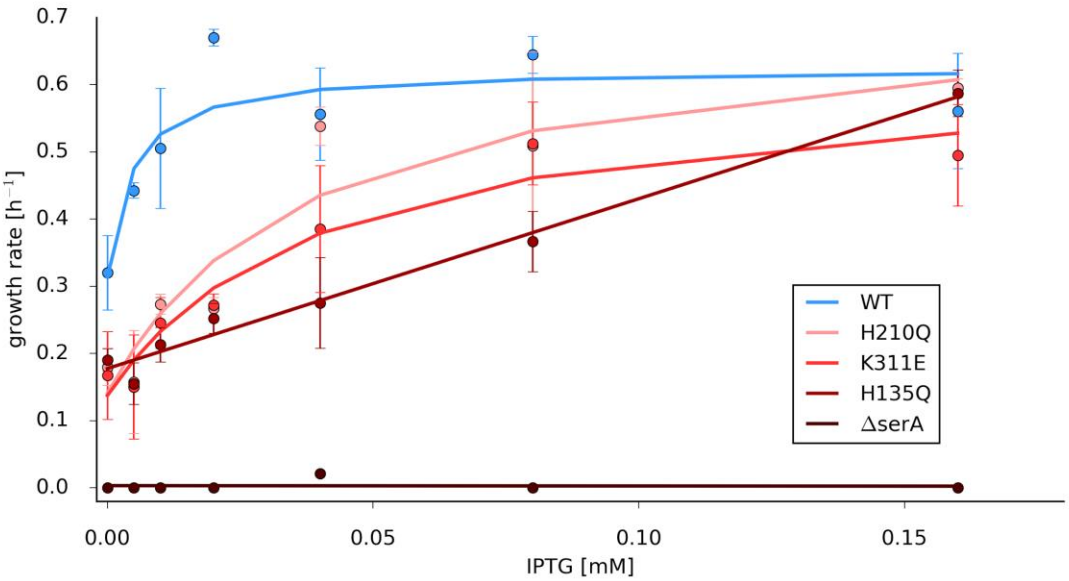

Supplementary Figure 5. The observed *serA* mutants show slower *in vivo* activity relative to the wild type enzyme. *ΔserA* strains were complemented with a plasmid carrying a wild type copy of *serA* or one of three *serA* mutated enzymes (H210Q, K311E and H135Q). Using different concentrations of IPTG (x-axis), the expression levels of all *serA* variants was altered and the growth rate of each strain was measured. As can be seen, strains carrying a *serA* mutant present slower growth rates relative to wild type. This growth rate deficit is alleviated when the expression level is high. Error-bars represent the standard errors from three biological replicates.

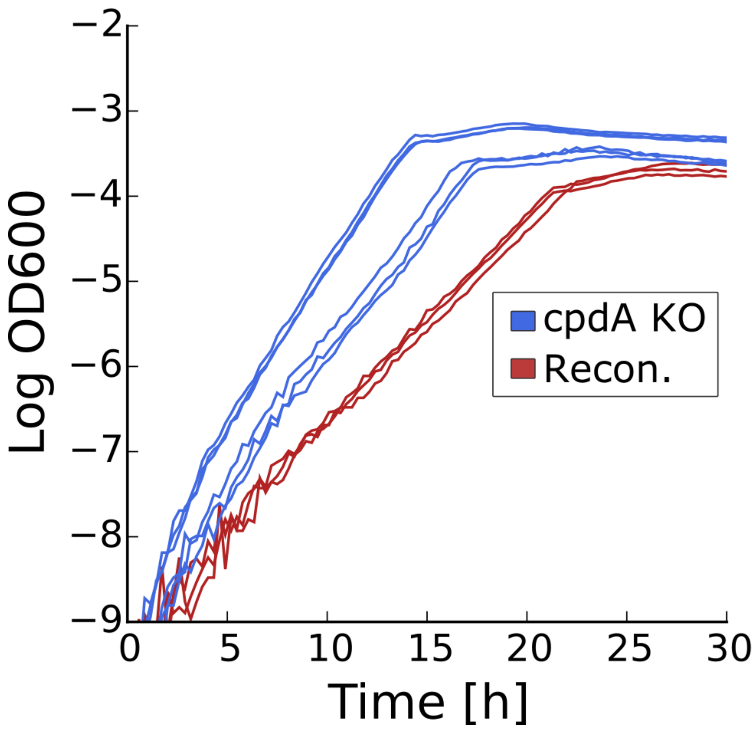

Supplementary Figure 6. Deletion of *cpdA* (*ΔcpdA*) is sufficient to complement the original *crp* mutation present in the reconstructed strain. A strain with *cpdA* deletion (Δ*cpdA*) on *crp*^{WT} background shows hemiautotrophic phenotype similar to the reconstructed strain (*crp* mutation (M190K)).

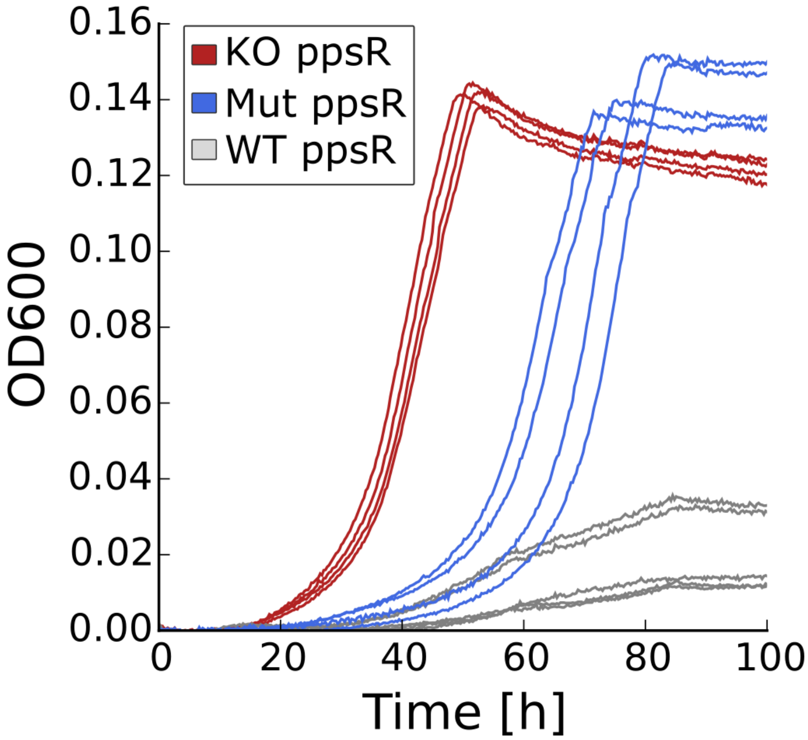

Supplementary Figure 7. Comparison of hemiautotrophic liquid growth of different *ppsR* variants. Hemiautotrophic liquid growth is detected in both *ppsR* leave-one-out colonies moved to liquid media (these strains contain all necessary mutations for hemiautotrophic growth except the *ppsR* mutation). According to sequencing data, cells containing wild-type *ppsR* (grey) are still the dominant fraction of the population. Strains containing mutant *ppsR* (blue; has E261D mutation) or *ppsR* knockout (red; Δ*ppsR*) exhibit significantly better growth rate and yield compared to strains containing wild-type *ppsR*.

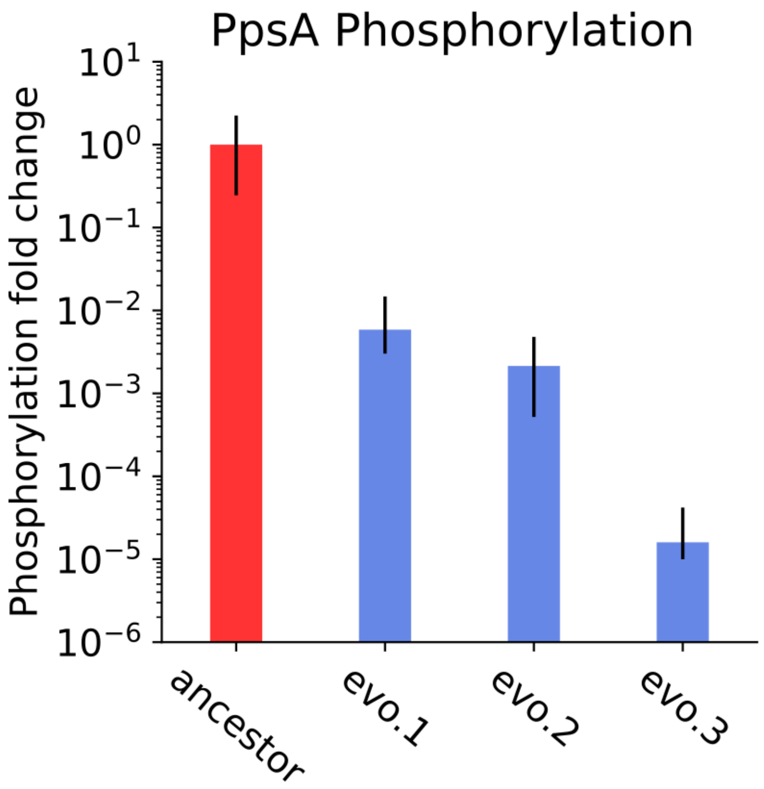

Supplementary Figure 8. The phosphorylation state of PpsA protein in evolved hemiautotrophic strains relative to the ancestral strain. Using targeted proteomics, we determine the phosphorylation state of the regulatory threonine residue of the PpsA protein in several different strains. All evolved strains contained a mutated *ppsR* gene: evolved 1 has 1bp deletion at base 834 in *ppsR* coding region; evolved 2 has A171V mutation in *ppsR*; evolved 3 has complete *ppsR* deletion. The ancestral strain has wild-type *ppsR*. The bar values represent the fold change (± S.E.; n=3) of the mean ratios of phosphorylated (inactive) to unphosphorylated (active) forms the regulatory threonine of PpsA relative to the ratio observed in the ancestral strain. Three biological replicates were used for each strain. Our analysis does not include copies of the protein which are phosphorylated in both histidine and threonine residues, as detailed in the Methods section.

Supplementary Table 1. Suppressor analysis for *pgi* WT. Details of the mutations observed in sequencing.

number	strain	mutation	note
-	pgi WT	• pgi mutation (G378C)	Original mutation -phosphoglucose isomerase
1		• pgi mutation (D185Y)	phosphoglucose isomerase
2		• pgi mutation Q394* (stop codon)	phosphoglucose isomerase
3		• pgi mutation (28bp deletion coding 228/1650)	phosphoglucose isomerase
4		• pgi mutation (13bp deletion coding 765/1650)	phosphoglucose isomerase
5		• pgm mutation (M269K)	phosphoglucomutase
6		• pgm mutation (M538L)	phosphoglucomutase
7		• pgm mutation (G46S)	phosphoglucomutase
8		• pgm mutation (H310Q)	phosphoglucomutase
9		• pgm mutation (Y518C)	phosphoglucomutase
10		• pgm mutation (V83E)	phosphoglucomutase

Supplementary Table 2. suppressor analysis for *serA* WT. Details of the mutations observed in sequencing.

number	strain	mutation	note
-	serA WT	• serA mutation (H210Q)	original mutation-<br>3-phosphoglycerate dehydrogenase
1		• serA mutation (D181N)	3-phosphoglycerate dehydrogenase- (NAD binding site)
2		• serA mutation (G294C)	3-phosphoglycerate dehydrogenase- (Nucleotide binding site)
3		• serA mutation (K311E)	3-phosphoglycerate dehydrogenase
4		• serA mutation (H135Q)	3-phosphoglycerate dehydrogenase
5		• prs/ispE (- 85/+66 +TAT insertion)	intergenic mutation
6		• Prs duplication extension (R105_A110 dup) -coding (310/948 nt)	Ribose-phosphate diphosphokinase-<br>The six amino-acid duplication that occurred in the evolved strain was extended into 12 amino-acid duplication
7		• Prs duplication extension<br>• nudE-IS element insertion -coding (226/561 nt).	• Ribose-phosphate diphosphokinase- coding (310/948 nt)<br>• ADP-sugar pyrophosphorylase- IS element insertion
8		• dnaE mutation (G1111R)	DNA polymerase III, α subunit

Supplementary Table 3. suppressor analysis for *prs* WT. Details of the mutations observed in sequencing.

number	strain	mutation	note
-	prs^{WT}	• prs mutation (R105_A110 dup)	original mutation-<br>Ribose-phosphate diphosphokinase
1		• prs mutation (Q134K)<br>• CpxA mutation (W184R)	• Ribose-phosphate diphosphokinase<br>• sensory histidine kinase
2		• prs mutation (E133Q)<br>• ydiV mutation (Q59K)<br>• **yehT(*G238V)<br>• **typA* (A118S)	• Ribose-phosphate diphosphokinase<br>• anti-FlhDC factor
3		• prs mutation (E133Q)<br>• typA mutation (A118S)<br>• **ydiV*(Q59K)<br>• **yehT* (G238V)	• Ribose-phosphate diphosphokinase<br>• ribosome-dependent GTPase
4		• cpxA(W184R)	sensory histidine kinase
5		• cpxA mutation (W184R),<br>• yhiJ/yhiL intergenic (- 41/+221) IS5 insertion	sensory histidine kinase
6		• yqiA(S12I)<br>• cpxA(W184R)<br>• pitA (M48R)	• Esterase<br>• sensory histidine kinase<br>• metal phosphate:H+ symporter
7		• gpp(2bp del-coding (133- 134/1485 nt))	pppGpp pyrophosphatase
8		• proA mutation (5 bp deletion coding 1041- 1045/1254 nt)<br>• proA mutation (1 bp deletion coding 1054/1254 nt)	glutamate-5-semialdehyde dehydrogenase
9		• proA mutation (A352P)	glutamate-5-semialdehyde dehydrogenase

		• yjiY/tsr mutation (IS5 insertion - 48/- 330)	
10		• yjiY/tsr mutation (IS5 intergenic insertion - 48/- 330)	
11		• yjiY mutation (+4 bp coding 8-11/2151 nt)	inner membrane protein - predicted transporter
12		• yjiY/tsr mutation (IS5 intergenic insertion - 148/- 230)	
13		• yjiY/tsr mutation (IS5 intergenic insertion - 45/- 333)	
14	prs^{WT}	• yjiY/tsr mutation (IS5 intergenic insertion - 48/- 330)<br>• yhiM/yhiN mutation (Intergenic IS5 insertion +58/+257)	
15		• pitA mutation (+11 bp duplication coding 1381/1500 nt)	metal phosphate:H+ symporter
16		• typA mutation (A118S),<br>• pitA mutation (+4 bp insertion coding 868-871/1500 nt),<br>• yjiY/tsr mutation (IS5 intergenic insertion - 45/- 333)	• ribosome-dependent GTPase<br>• metal phosphate:H+ symporter
17		Removal of three sections by IS elements:<br>• yhiM-pitA,<br>• dtpB-rlmJ<br>• gor-dinQ	
18		• yhiM/yhiN mutation (Intergenic IS5 insertion +58/+257)<br>• yjbE/aqp (intergenic-369/+126 new GC)	

***note:** mutations below the 50% threshold that may also be connected

Supplementary Table 4. suppressor analysis for *crp* WT. Details of the mutations observed in sequencing.

number	strain	mutation	note
-	crp WT	• crp mutation (M190K)	original mutation-<br>CRP transcriptional dual regulator
1		• crp mutation (L135M)	CRP transcriptional dual regulator
2		• crp mutation (A145E)	CRP transcriptional dual regulator
3		• crp mutation (T141R)	CRP transcriptional dual regulator
4		• cpdA 120bp duplication (coding 491/948)	cAMP phosphodiesterase
5		• cyaA mutation (K421N)	adenylate cyclase
6		• cyaA mutation (K421N)<br>• pitA mutation (IS4 (–) +12 bp) in coding	• adenylate cyclase<br>• metal phosphate:H+ symporter
7		• cyaA mutation (K421N),<br>• pitA mutation (L10*)	• adenylate cyclase<br>• metal phosphate:H+ symporter
8		• cyaA mutation (Y394H)	adenylate cyclase
9		• cyaA mutation (Y394H)<br>• pitA mutation (IS5) in coding	• adenylate cyclase<br>• metal phosphate:H+ symporter
10		• lgoR (S291*)<br>• arcA mutation(R163C)<br>• pstS (W304*)	• predicted DNA-binding transcriptional regulator<br>• transcriptional dual regulator<br>• phosphate ABC transporter - periplasmic binding protein
11		• lgoR (S291*)<br>• arcA mutation(R163C)<br>• fabH (coding (878/954 nt) missing T)	• predicted DNA-binding transcriptional regulator<br>• transcriptional dual regulator<br>• β-ketoacyl-ACP synthase III
12		• gdhA mut (G89V),<br>• yhiM(IS5-coding 286/1053 nt),	• glutamate dehydrogenase

		• serA/rpi intergenic mut (- 162/+94 IS5)	• inner membrane protein with a role in acid resistance
13		• yhiM (IS5-coding 206/1053 nt),<br>• epd/yggC intergenic insertion (- 136/+149 new T)	inner membrane protein with a role in acid resistance

Supplementary Table 5. suppressor analysis for *ppsR* WT. Details of the mutations observed in sequencing.

number	strain	mutation	note
-	ppsR WT	• ppsR large deletion (Δ810)	original mutation- PEP synthetase regulatory protein
1		• ppsR mutation- 1bp deletion (coding 440/834)	PEP synthetase regulatory protein
2		• ppsR mutation- 18bp duplication (coding 374/834)	PEP synthetase regulatory protein
3		• ppsR mutation- transposon insertion 1 (coding 271/834)	PEP synthetase regulatory protein
4		• ppsR mutation- transposon insertion 2 (coding 266/834)	PEP synthetase regulatory protein
5		• ppsR mutation- transposon insertion 3(coding 58/834)	PEP synthetase regulatory protein
6		• ppsR mutation- transposon insertion 4 (coding 12/834)	PEP synthetase regulatory protein
7		• ppsR mutation (E261D)	PEP synthetase regulatory protein

Supplementary Table 6 – Deletion list and primers used for verification of genomic modification

strain	Gene deletion	adjacent mutation	primer F	primer R
ancestral	gpmA *(JW0738)*		TTACGTCAACTGGCGAATGC	CTCGTCATGAGGGCTTTATC
ancestral	gpmM (JW3587)		GGTAACAACTCCCGACGTAG	GGCGATGTCAGCCTGAATAG
ancestral	pfkA (JW3887)		AGGGAGGGTAAACGGTCTATG	CTTGCGGGTATATGTTGAGGG
ancestral	pfkB (JW5280)		TTAGCGTCCCTGGAAAGGTAAC	TCCCTCATCATCCGTCATAGTG
ancestral	aceB-A-K		CTTACCTCAGGCACCTTCGG	GGTCACCGGGTTATTGCTGA
ancestral	Zwf (JW1841)		GCAGGATGATTCACAACGCG	GCCTGTGTGCCGTGTTAATG
Reconstructed	ppsR (JW1693)		CATCATTCATGCCGAGTTGG	AGTACGGAGTTCGTCAGTTC
Reconstructed	ychH/dauA (JW1196 /JW5189)	prs	CAAGGTGTTCAGGCGTTTATTT	TACTTGATGCTGGTGGTCTTG
Reconstructed	yjbI (JW3998)	pgi	GCCTGGGATCGACATCTGCC	GGGCATCACCGTCCAGGATG
Reconstructed	yqfA (JW2867)	serA	CGCATCAGGCATTTATCGCC	CGCTGGATACGCTGACTGAA
Reconstructed	chiA (JW3300)	crp	ATTGCTGGAACGAGTGAGGG	TGCGTAGGACTTTTGTTTTGCA

Note: The Reconstructed strain contains both ancestral deletion and 'reconstructed' ones.

Supplementary Table 7- MASC primers

name	sequence	amplicon size
serA masc F-MUT	CCA TCA TAT TTT TGG TGG ACG GAT TCT CTG GTA CT	169
serA masc F-WT	CCA TCA TAT TTT TGG TGG ACG GAT TCT CTG GTA CA	
serA masc REV	TGG GCA TTC TGG CTG AAT CGC TG	
pgi masc F-MUT	GGA TTA CCA GAC TGG CCC GAT TAT CTG GT	267
pgi masc F-WT	GGA TTA CCA GAC TGG CCC GAT TAT CTG GG	
pgi masc REV	ACG TAG TCA AGC GTT GCC GGA TCT	
crp masc F-MUT	GTG AAA CCG TGG GAC GCA TTC TGA AGA A	379
crp masc F-WT	GTG AAA CCG TGG GAC GCA TTC TGA AGA T	
crp masc REV	TAG CTG TGT CAG CAA GCT ACA GGT GG	
malT masc F_MUT	GCG GAT GGA TGA TAC CGG CGA GA	443
malT masc F-WT	GCG GAT GGA TGA TAC CGG CGA GT	
malT masc REV	GGG CGC GCA GAG CGT TAA ATT CTG	
xylA masc F-MUT	CGT CGC GTG GTT AGC TTC AAT GTT CAG TTG	550
xylA masc F-WT	CGT CGC GTG GTT AGC TTC AAT GTT CAG TTT	
xylA masc REV	CCA CAA GTT ACA TGT GCC ATT TTA TTG CTT CCA CG	
